# Allosteric Modulators of GABA_B_ Receptors: Mechanism of Action and Therapeutic Perspective

**DOI:** 10.2174/157015907781695919

**Published:** 2007-09

**Authors:** Jean-Philippe Pin, Laurent Prézeau

**Affiliations:** CNRS-UMR 5203, Montpellier, France; INSERM-U661, Montpellier, France; Univ. Montpellier 1, Montpellier, France; Univ. Montpellier 2, Montpellier, France; Institut of functional Genomics (IGF), Department of Molecular Pharmacology, Montpellier, France

**Keywords:** Baclofen, anxiety, drug addiction, allosteric modulators, class C GPCRs.

## Abstract

γ-aminobutyric acid (GABA) plays important roles in the central nervous system, acting as a neurotransmitter on both ionotropic ligand-gated Cl^-^-channels, and metabotropic G-protein coupled receptors (GPCRs). These two types of receptors called GABA_A_ (and C) and GABA_B_ are the targets of major therapeutic drugs such as the anxiolytic benzodiazepines, and antispastic drug baclofen (lioresal®), respectively. Although the multiplicity of GABA_A_ receptors offer a number of possibilities to discover new and more selective drugs, the molecular characterization of the GABA_B_ receptor revealed a unique, though complex, heterodimeric GPCR. High throughput screening strategies carried out in pharmaceutical industries, helped identifying new compounds positively modulating the activity of the GABA_B_ receptor. These molecules, almost devoid of apparent activity when applied alone, greatly enhance both the potency and efficacy of GABA_B_ agonists. As such, in contrast to baclofen that constantly activates the receptor everywhere in the brain, these positive allosteric modulators induce a large increase in GABA_B_-mediated responses only WHERE and WHEN physiologically needed. Such compounds are then well adapted to help GABA to activate its GABA_B_ receptors, like benzodiazepines favor GABA_A_ receptor activation. In this review, the way of action of these molecules will be presented in light of our actual knowledge of the activation mechanism of the GABA_B_ receptor. We will then show that, as expected, these molecules have more pronounced *in vivo* responses and less side effects than pure agonists, offering new potential therapeutic applications for this new class of GABA_B_ ligands.

## INTRODUCTION

As one of the major neurotransmitters in the brain, γ -amino-butyric acid (GABA) plays critical roles in brain development and physiology. By activating GABA_A_ receptors, which are Cl^-^-gated channels, this neurotransmitter prevents neuronal depolarization, and as such controls the transmission of excitatory signals. In young animals, these GABA_A_ receptors generate instead excitatory responses, and replace the glutamatergic system not yet fully established. Controlling GABA_A_ receptor activity soon appeared as an interesting way for the treatment of brain dysfunction. This led to the discovery of benzodiazepines that allosterically enhance GABA_A_ receptor activation by acting at a site distinct from the GABA binding site. These positive modulators act by increasing GABA affinity and potency, and by facilitating Cl^-^-channel opening, and are widely used for the treatment of insomnia, anxiety and epilepsies.

GABA also acts on G protein-coupled GABA_B_ receptors [6]. These receptors limit neurotransmitter release at many synapses, including most GABAergic and glutamatergic ones, by inhibiting at least Ca^2+^-channel opening. They are also located in post-synaptic elements where they activate G protein-regulated inward-rectifying K^+^-channels (GIRK channels) [42]. These receptors were pharmacologically identified in the early 80's, being selectively activated by baclofen (β p-chlorophenyl-GABA) [31], a molecule that is used for the treatment of spasticity in multiple sclerosis patients due to its muscle-relaxant properties [9]. The GABA_B_ receptors are also responsible for most effects of the drug of abuse gamma-hydroxybutyrate (GHB) that acts as a GABA_B_ partial agonist at high doses [34, 48]. GABA_B_ agonists also demonstrated a number of beneficial effects both in animals and in humans [5]. Indeed, activation of GABA_B_ receptors exerts analgesic/antinociceptive effects in animal models of chronic inflammation and neuropathy (see [5]), suppresses drug seeking behavior [15], and has some anxiolytic activity both in animal models and in human [17]. However, undesired side effects such as hypothermic and sedative effects, greatly limits the use of GABA_B_ agonists in therapeutics [5]. Moreover, tolerance to baclofen chronic treatment is well established [44]. In addition to agonists, GABA_B_ antagonists were also shown to have potential therapeutic effects, such as antidepressant activity [17], cognition improvement [22], and beneficial effects in rat models of absence epilepsy [53].

GABA_B_ receptors have therefore been used as a target in high throughput screening strategies with the aim at identifying new ligands acting at this receptor. As already well documented for the related metabotropic glutamate (mGlu) receptors [26, 28], this strategy leads to the discovery of allosteric modulators acting at the GABA_B_ receptor [76, 77]. In contrast to mGluRs for which both positive and negative (non-competitive antagonists) were identified, only positive allosteric modulators (PAMs) have been described so far for the GABA_B_ receptor. These compounds display no or very partial agonist activity, but enhance both the potency and efficacy of GABA_B_ agonists. As such, these molecules appear as a better alternative to GABA_B_ agonists, allowing the specific enhancement of GABA_B_ receptor activity when and where needed, and as such, are less prone to tolerance in contrast to the pure agonists that constantly activate the receptor in any region where it is expressed.

In the present chapter, we aimed at describing the mechanism of action of the identified allosteric modulators of the GABA_B_ receptor. We will first describe our current knowledge of the functioning of this complex receptor (for reviews see [2, 65]). We will then highlight the potential new therapeutic possibilities offered by these molecules, as based on the recent preclinical studies reported in the literature.

## STRUCTURE AND ACTIVATION MECHANISM OF THE GABA_B_ RECEPTOR

**Fig. (1):**
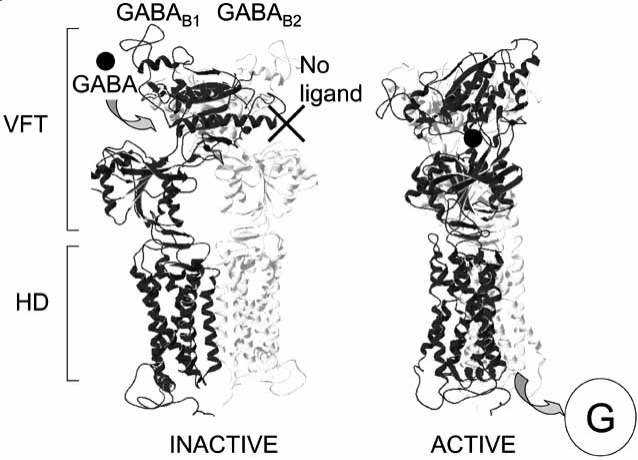
Expected structure of the heterodimeric GABA_B_ receptor in its inactive (left) and active (right) states. This receptor is made of two homologous subunits, GABA_B1_ (in the front, in black) and GABA_B2_ (in the back, in light grey). Each subunit is made of two main domains, the extracellular Venus Flytrap domain (VFT) and the heptahelical domain (HD). GABA and other orthosteric GABA_B_ ligands are known to bind in the GABA_B1_ VFT. No known ligand bind at the equivalent site in GABA_B2_. Only the GABA_B2_ HD appears to be responsible for G-protein coupling. These images were made using the coordinates of the dimer of mGlu1 VFTs in the inactive empty state (left) and those of the active Glu occupied state (right) [43], in association with a dimer of HD generated based on the proposed model of the dimer of rhodopsin [47].

The GABA_B_ receptor is part of the class C of GPCRs that also includes the mGlu, the Ca^2+^-sensing, and the sweet and umami taste receptors among others [64]. These receptors are dimers, either homodimers linked by a disulphide bond (mGlu and Ca^2+^-sensing receptors), or heterodimers made of two similar, but distinct subunits (the GABA_B_ and taste receptors). Indeed, the GABA_B_ receptor was the first GPCR to be identified that requires two distinct subunits to function: the GABA_B1_ and GABA_B2_ subunits [33, 36, 79] (Fig. **1**). Although the GABA_B1_ subunit was soon shown to bind all known GABA_B_ ligands (both agonists and antagonists), this protein did not form a functional GABA_B_ receptor when expressed alone [35]. Only when GABA_B1_ was co-expressed with the homologous GABA_B2_ subunit was a functional GABA_B_ receptor observed, either in cell lines or in cultured neurons. The GABA_B_ dimeric entity was confirmed in native tissue [36]. Indeed, both GABA_B1_ and GABA_B2_ mRNAs are co-localized in most brain regions. Second, both proteins are found in the same neurons, even in the same subcellular compartments as observed at the electron microscopic level. Moreover, co-immunoprecipitation of GABA_B1_ with a GABA_B2_ antibody could be demonstrated from brain membranes. Eventually, mice lacking either GABA_B1_ or GABA_B2_ share very similar phenotypes, and none of the known GABA_B_-mediated responses could be measured in either mice [66, 69]. Although unusual baclofen-mediated inhibition of GIRK channels could be observed in mice lacking GABA_B2_, it is still not known whether this represents a natural response, or is the consequence of the absence of the GABA_B2_ subunit. Taken together, these data demonstrate that the assembly between these two proteins is required to get a functional GABA_B_ receptor in native tissues.

When expressed alone, the GABA_B1_ subunit is mostly retained in the endoplasmic reticulum (ER), both in transfected cell lines and in neurons [16]. This is due to the presence of an intracellular retention signal (RXR) located in its intracellular tail that constitutes a binding site for the coat protein-I complex (COPI) [8, 10, 54, 61]. COPI is known to target back to the ER proteins that reached the *cis*-Golgi, therefore preventing their trafficking through the Golgi and their targeting to the cell surface. COPI binding to the RXR motif of GABA_B1_ is prevented by GABA_B2_ thanks to a direct interaction between the intracellular tails of these two subunits through a coiled-coil interaction [8, 10, 54, 61]. Such a system is assumed to control the trafficking to the cell surface of correctly assembled GABA_B_ heterodimers.

Each GABA_B_ receptor subunit is made of two main domains: a large extracellular domain structurally similar to bacterial periplasmic amino-acid binding proteins often called a Venus Flytrap domain (VFT) [24], linked to a 7 transmembrane domain (the heptahelical domain (HD)) typical of all GPCRs (Fig. ** 1**). Besides these common features, most class C GPCRs, except the GABA_B_ receptor subunits, possess a cystein-rich domain that interconnects, both physically and functionally, the VFT and the HD in mGlu receptors [68]. A third domain composed of two short consensus repeats, also known as Sushi domains, is found at the N-terminus of the GABA_B1a_ splice variant but in the GABA_B1b_ variant, [30, 35]. These Sushi domains are responsible for the specific targeting of the GABA_B1a_ receptor in nerve terminals of glutamatergic neurons [78].

The VFT domain of class C GPCRs contains the binding site for agonists and competitive antagonists (the orthosteric ligands). In the case of the GABA_B_ receptor, GABA and all other orthosteric ligands bind to the GABA_B1_ VFT only, notably by interacting with Ser246 and Glu465 (nomenclature based on the rat GABA_B1a_ sequence) [24, 39, 52]. Indeed, mutational and evolution analyses of the GABA_B2_ VFT suggest that no natural ligand binds in this domain [39].

Although GABA binds in the GABA_B1_ VFT, it is now well demonstrated that the GABA_B2_ HD is responsible for G-protein activation (Fig. ** 1**). Indeed, a mutated receptor dimer with two GABA_B2_ HDs is functional, whereas a mutated receptor with two GABA_B1_ HDs does not [23]. Moreover, mutations in either the second or third intracellular loop of GABA_B2_ suppress G-protein activation whereas the equivalent mutations in GABA_B1_ do not [21, 29, 67]. Finally, a recent study identified an Arg residue at the bottom of TM3 conserved in most class C GPCRs that plays a critical role in G-protein activation [4]. This Arg may possibly play a role similar to that of the conserved D/ERY motif of class A GPCRs. Of interest, this Arg is found in the GABA_B2_, but not in the GABA_B1_, further highlighting the pivotal role played by GABA_B2_ in G-protein activation.

How can agonist binding in GABA_B1_ VFT activate the GABA_B2_ HD? Much information to answer that question came from the solved crystal structure of the mGlu1 VFT dimer with and without bound agonist or antagonist [43, 73]. These structures revealed that agonist binding in the VFT stabilizes a closed conformation that is also associated with a major change in the relative orientation of the two VFTs in the dimer (Fig. ** 1**). This relative movement is expected to induce a relative movement of the HDs, a proposal that is consistent with FRET studies [72]. Of interest, although both HDs in a mGlu homodimer are identical, this process leads to the active state of only one of them [32], likely because a single G-protein can interact at a time with such dimeric entities [19]. This model perfectly fits with all mutational analysis of GABA_B_ receptor functioning. Indeed, the closure of the GABA_B1_ VFT has been shown to be responsible for GABA_B_ receptor activation [40], and such a closure activates GABA_B2_ HD whether it is part of the associated subunit (like in the wild-type heterodimer) or linked to the GABA_B1_ VFT [23]. Moreover, point mutations introduced into either the GABA_B1_ VFT or the GABA_B2_ HD were found to increase constitutive activity of this receptor, consistent with these two domains playing a critical role in receptor activation [56].

In summary, the GABA_B_ receptor is a complex allosteric protein made or four main domains working "de concert" to allow GABA binding in the VFT of one subunit (GABA_B1_) to activate the HD of the associated subunit (GABA_B2_), likely through relative movement between these domains (Fig. ** 1**). As we will see now, such a complex structure offers a number of possibilities to modulate GABA_B_ receptor function.

## ALLOSTERIC MODULATORS OF THE GABA_B_RECEPTOR: PROPERTIES AND MECHANISM OF ACTION

Early studies following the molecular characterization of the GABA_B_ receptor heterodimer indicated that Ca^2+^ ions act as enhancers of this receptor [80]. Indeed, few hundred micromolar of Ca^2+^ increased the potency of GABA in stimulating GTPγ S binding or G-protein activation measured in second messenger assays [25]. This effect is observed both with the recombinant and the native receptor [25], even in post-morten human tissues [58], and results from a direct increase in GABA affinity. Of interest, this effect of Ca^2+^ was not observed with baclofen, suggesting that the chlorophenyl group of baclofen prevents the action of Ca^2+^ ions, pointing to the possibility that Ca^2+^ directly binds within the GABA binding site in the GABA_B1_ VFT. This was further validated using site directed and 3D modeling studies [25]. According to the expected physiological Ca^2+^ concentration range, the GABA_B_ receptor is expected to be always potentiated under physiological condition. Only under pathological conditions, when the extracellular Ca^2+^ concentration reaches values as low as few micromolar, can this effect disappear. Whatever, these data revealed that it is possible to positively modulate GABA_B_ receptor function with small molecules.

Few years before this observation, a number of allosteric modulators of the other class C GPCRs, and especially mGlu receptors, were identified, including both negative and positive allosteric modulators [26, 28] (see this issue). The negative modulators first identified for mGlu1 and mGlu5, were found to inhibit in a non-competitive manner the activity of the receptors, and to display in most cases inverse agonist activity [12, 62]. In contrast, PAMs were found to have no, or weak agonist activity when applied alone, but to greatly enhance both the potency and the efficacy of agonists [41, 57]. Both types of modulators were found to bind in a cavity within the HD, contacting residues of TM3, TM5 TM6 and TM7, therefore at a site clearly distinct from the glutamate binding site located in the VFT. Residues that constitute this binding site differ between receptor subtypes, such that most modulators identified so far, either positive or negative, were found to be highly subtype selective, in contrast to the orthosteric ligands that usually do not discriminate between mGlu receptors from the same group [26, 28].

Taken together, these data indicated that compounds interacting in the HD of class C GPCRs could allosterically modulate their activity, and these compounds had three main advantages: 1) original chemical structures, different from that of the orthosteric ligands, usually poly-cyclic with a good bioavailability, more prone to chemical modifications, and in agreement with the Lipinski's rules for drug-likelyness; 2) much higher selectivity among related sequences; and 3) a good respect of the biological activity of the receptors, especially for the PAMs that facilitate agonist action, and therefore enhance receptor activation when and where needed physiologically.

**Fig. (2):**
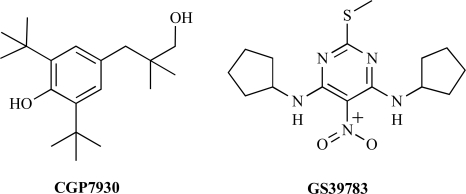
Structure of the two PAMs identified for the GABA_B_ receptor.

These observations lead a number of pharmaceutical companies to search for new GABA_B_ modulators using high throughput functional assays. So far, only 2 PAMs have been reported in the literature (2,6-Di-tert.-butyl-4-(3-hydroxy-2,2-dimethyl-propyl)-phenol (CGP7930) and N,N'-Dicyclopentyl-2-methylsulfanyl-5-nitro-pyrimidine-4,6-diamine (GS39783) and some of their derivatives) [76, 77] (Fig. ** 2**), and some others have been reported in patents [49-51]. Aryl-alkylamine (such as fendiline), amino acids like phenylalanine, leucine and isoleucine as well as dipeptides have also been shown to enhance GABA_B_ receptor activity in brain slices [14, 37, 38]. However, others reported that fendiline inhibits, rather than mimick, the effect of CGP7930 [58], and Urwyller and colleagues show that the effect of Aryl-alkylamine and amino acids are rather indirect, and do not result from a direct PAM action on the receptor itself [74].

**Fig. (3):**
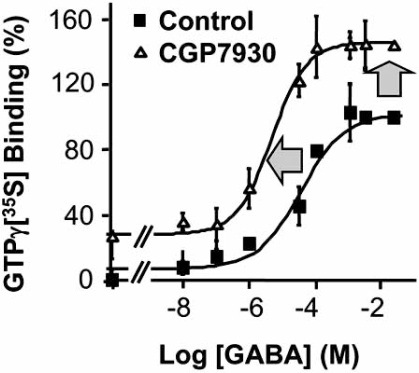
The PAMs increase both the potency and efficacy of GABA on the GABA_B_ receptor. Data were obtained from membranes prepared from HEK 293 cells expressing GABA_B1_, GABA_B2_ and the Gαo proteins. GTPγS binding was measured in the presence of the indicated concentration of GABA with (open triangles) or without (closed squares) 100 µM CGP7930. This figure is adapted from [3].

CGP7930 and GS39783 were found to enhance agonist potency as well as efficacy on recombinant GABA_B_ receptors in various assays (Fig. ** 3**), on both human and rat receptors [20, 76, 77]. GS39783 was also shown to be active on fish and chicken receptors, but not on the Drosophila one [20], and CGP7930 enhances GABA affinity on the bullfrog receptor [1], demonstrating a good conservation of the allosteric site in vertebrates. In the GTPγ S binding assay CGP7930 and GS39783 increase GABA potency by 5-10 fold, and increase the maximal effect from 1.5 to up to 2 fold, with potencies ranging from 3 to 5 µM, depending on the agonist concentration. The same positive allosteric effect was also observed when coupling of the GABA_B_ receptor to GIRK channels was measured in *Xenopus* oocytes [76, 77], or when the coupling of the receptor to phospholipase C was made possible with recombinant chimeric Gqi/o proteins [3, 76]. Very similar enhancing effects were observed with all three well known GABA_B_ receptor agonists, GABA, baclofen and APPA. Of interest, the PAMs largely increased the efficacy of partial agonists like CGP47656 to make it a full agonist, with a similar maximal effect as that of GABA. Moreover, among 7 competitive antagonists, two (CGP35348 and 2-OH-saclofen) became partial agonists [75].

CGP7930 and GS39783 increased agonist affinity as measured with [^3^H]-APPA or through the displacement of radio-labeled antagonists [75-77]. However, the increase in affinity (2 fold) is lower than the measured increase in potency. In agreement with the allosteric potentiator further stabilizing the closed state of the GB1 VFT, a decrease in both the ON and OFF binding rates of agonists was observed, as well as a slight decrease in the affinity of most antagonists [63]. Only the affinities of the antagonists CGP35348 and 2-OH-saclofen that became partial agonists in the presence of the PAMs, were increased [75].

The two identified GABA_B_ PAMs show no or only slight agonist activity when applied alone in most assays, both in recombinant systems, and in native preparations [20, 59, 76, 77]. However, partial agonist activity of CGP7930 could be observed when IP production was measured in HEK293 cells co-expressing the GABA_B_ receptor and the chimeric G-protein Gqi9 [3]. This was not the consequence of endogenous agonists in the preparation since the competitive antagonists could not fully inhibit the effect of CGP7930. Although this likely results from the over-expression of the receptor and/or its coupling to non natural G-proteins, these data show that CGP7930 acts by stabilizing the receptor in its active state, an effect that is greatly favored in the presence of agonist. In agreement with this proposal, point mutations in the GABA_B2_ subunit were found to convert GS39783 from a pure PAM into a partial agonist, even though these mutations did not appear to generate a constitutively active receptor [20].

To identify the mode of action of CGP7930, Binet and colleagues studied its effect on various combinations of wild-type and chimeric GABA_B_ subunits, and took advantage of the agonist activity of this molecule in their assay [3]. This study revealed that the GABA_B2_ HD was required and sufficient for CGP7930 action. Indeed, CGP7930 was found to activate GABA_B2_ subunit expressed alone, as well as a truncated version of this subunit corresponding to the HD only. Dupuis and colleagues make use of the absence of effect of GS39783 on the *Drosophila* GABA_B_ receptor to identify its mechanism of action using chimeric drosophila/rat subunits [20]. They also bring further evidence for GS39783 acting in the HD of GABA_B2_. These authors also tried to identify the residues within the GABA_B2_ HD that interact with GS39783. Although no such residues were identified, mutations in TM6 were found to convert the modulator into an agonist, suggesting that the mutated residues are involved in stabilizing the GABA_B2_ HD into its inactive conformation. Residues of the extracellular side of TM7 were also found to decrease GS39783 efficacy, but not its potency, suggesting that these residues are involved in the allosteric coupling between the HD and the VFT in the GABA_B_ receptor. It is quite surprising that among the large number of mutants generated, none affected GS39783 potency. More work is therefore needed to better understand the mode of action of GABA_B_ PAMs at the atomic level.

**Fig. (4):**
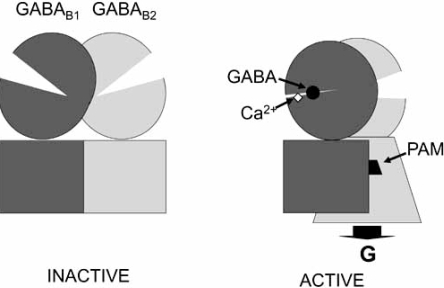
Schematic view of the mechanism of action of GABA_B_ PAMs as based on the proposed activation mechanism of this heterodimeric receptor. By stabilizing the closed state of the GABA_B1_ VFT (dark grey), Ca^2+^ increases GABA affinity and potency. By stabilizing the active conformation of the GABA_B2_ HD, small molecule PAMs increase both the potency and efficacy of agonists. The absence of agonist activity of these molecule may be due to their difficulty in promoting the relative movement between the subunits, a change that is proposed to play a critical role in receptor activation.

In summary, GABA_B_ receptor activation is due to the closure of the GABA_B1_ VFT, that likely results in a relative movement of one subunit compare to the other. This new conformation of the heterodimer stabilizes the active conformation of the GABA_B2_ HD that promote the GDP-GTP exchange in the associated G-protein (Fig. ** 4**). As such there are two possibilities to enhance receptor activity. By further stabilizing the close state of GABA_B1_ VFT, as likely does Ca^2+^, or stabilizing the active conformation of GABA_B2_ HD as do CGP7930 and GS39783 (Fig. ** 4**). In the absence of agonist, these later compounds may still bind in the GABA_B2_ HD, but may not lead to the relative movement between the subunits, preventing them from being agonists (Fig. ** 4**).

## *IN VIVO* EFFECT OF POSITIVE ALLOSTERIC MODULATORS (PAMS)

### Action of GABA_B_ PAMs on Native Receptors

Soon after their identification and characterization on recombinant GABA_B_ receptors, the PAMs were shown to be effective on native receptors. This is nicely illustrated with the increase in agonist affinity and the potentiation of baclofen stimulated GTPγS binding by both CGP7930 and GS39783 in rat cortical membranes [75-77], as well as in human fontal cortex membranes [58]. Measurement of either the inhibition or stimulation of cAMP formation in native brain membranes and *in vivo* also confirmed the PAM activity of these two compounds at the native GABA_B_ receptor [27, 59]. When examined in brain slices, these compounds potentiated GABA_B_ receptor action on synaptic transmission. GS39783 suppresses the paired pulse inhibition of population spikes recorded on hippocampal CA1 pyramidal cells, an effect that likely results from the potentiation of the action of ambiant GABA at pre-synaptic GABA_B_ receptors located on GABAergic terminals [77]. The other GABA_B_ enhancer CGP7930 enhances baclofen-induced depression of dopaminergic neurons in the ventral tegmental area [14] and the GABAergic synaptic transmission in the CA1 area of the hippocampus [13]. Surprisingly, no significant effect on excitatory synaptic transmission in hippocampal CA1 network was observed [13] with CGP7930. It is proposed that this may result from a differential effect of this enhancer on the autoreceptors located in GABAergic terminals, and the heteroreceptors located in glutamatergic terminals. Although GABA_B1a_ and GABA_B1b_ splice variants have been shown to be differentially distributed in these two types of terminals [78], CGP7930 was found to be equally active on both recombinant receptors. Further studies are therefore required to clarify this issue.

Most importantly, both CGP7930 and GS39783 were found to pass the blood brain barrier when injected i.p. (or even when given orally in the case of GS39783) allowing the examination of their behavioral effects* in vivo*. Indeed, GS39783 decreased cAMP formation *in vivo* in the striatum only when co-administered orally with a threshold concentration of baclofen [27]. *In vivo* efficacy of CGP7930 was also illustrated by its marked enhancement of the sedative and hypnotic effect of both baclofen and GHB in DBA mice [11]. Due to the original mechanism of action of these PAMs, it was therefore of interest to examine whether such compounds have different effects than the GABA_B_ agonist baclofen.

### Differential Effects of PAMs and Agonists

**Fig. (5):**
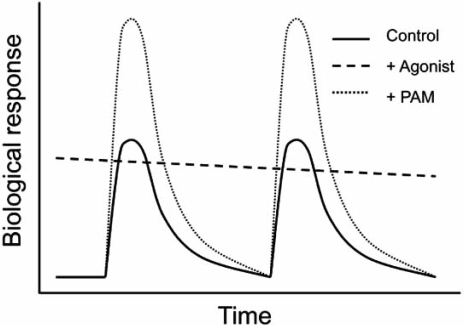
Major difference in the effect of agonists and PAMs acting at the GABA_B_ receptor. Scheme illustrate biological responses resulting from the physiological activity of the GABA_B_ receptor. In plain thick line is the response mediated under control condition. In the presence of a pure agonist, the receptor is always activated, with a decline resulting from the desensitization of the system and tolerance to the drug (dashed thick line). In contrast the PAM does not activate the system unless GABA is released close to the receptor. As such, the PAM enhances the response mediated by physiologically released GABA, enhancing the GABA mediated response, WHEN and WHERE needed (dashed thin line).

Although baclofen is being used in the treatment of spasticity for multiple sclerosis patients, its myorelaxant, sedative, cognitive and hypothermic effects limit its use in a number of other pathologies. In contrast to baclofen and other GABA_B_ agonists that activate constantly and everywhere the receptor, PAMs are expected to enhance receptor activity only WHEN and WHERE needed physiologically (when and where GABA is produced to act on the GABA_B_ receptor) (Fig. ** 5**). As such, differential effects of PAMs and agonists were expected. Indeed, GS39783 given alone did not display sedative, cognitive, myorelaxant activities [18]. However, sedative effects were reported for CGP7930 at high doses [46]. No effect of GS39783 on body temperature was also observed [18]. This document the general idea that PAMs could be a better alternative to baclofen for the treatment of pathologies in which such side effects are not desired. Of interest, as described in more details bellow, the PAMs display more pronounced anxiolytic effects than GABA_B_ agonists and keep most of the known positive actions of baclofen (Table ** 1**).

### GABA_B_ PAMs as Potential New Anxiolytics

The GABA system is well known to be involved in anxiety, as illustrated by the effect of benzodiazepines. However, the involvement of the GABA_B_ receptor remained elusive for a long time due to the difficulty in assessing the effect of baclofen because of its above mentioned side effects. Anxiolytic effects of baclofen were however observed in some specific tests in rats, and also in humans [5, 17]. The generation of knockout mice deleted of either the GABA_B1_ or the GABA_B2_ gene confirmed a role of GABA_B_ receptor in anxiety [17], as illustrated in several tests such as the light-dark box, the elevated plus maze or the elevated zero maze [18, 55]. In these same tests, the GABA_B_ PAM GS39783 show strong anxiolytic activity, in contrast to baclofen [18, 55]. GS39783 was also efficient in reducing stress-induced hyperthermia [18], a test that could not be performed with baclofen due to its hypothermic action. Of most interest, the anxiolytic effect of GS39783 could still be observed after three weeks of treatment, demonstrating an absence of tolerence [55]. Moreover, no synergy with alcohol was observed [17]. As such, GABA_B_ PAMs appear as a new class of anxiolytics that lack the side effects of the commonly used benzodiazepines.

### GABA_B_ PAMs for the Treatment of Drug Addiction

The GABA_B_ receptor is known for its role in modulating the reinforcing effect of abused drugs such as cocaine, heroin, alcohol, amphetamine and nicotine [15]. In rats, baclofen decreases self-administration of such drugs, and preclinical studies further indicated the potential of baclofen for the treatment of cocaine, alcohol and nicotine dependence. In support of these effects, baclofen attenuates the activation of limbic regions resulting from cocaine-associated cues as revealed by neuroimaging in humans [7]. However, the use of baclofen as a therapeutic strategy for these indications is limited due to its side effects. The effect of GABA_B_ PAMs on drug dependence and reinforcement has therefore been studied recently as a potential alternative to baclofen.

Both CGP7930 and GS39783 were found to inhibit cocaine self-administration in rats responding to different schedule of reinforcement [71]. Moreover, GS39783 inhibits the reward-facilitating effect of acute cocaine administration, as assessed by the reward threshold in intracranial self-stimulation paradigm [70]. The positive action of GS39783 on cocaine addiction is further supported by the inhibition of most biochemical and behavioral effects of acute and chronic cocaine treatment [45]. These include increased locomotor activity, up regulation of cAMP-response-element-binding protein (CREB) and phosphorylation of dopamine- and cAMP-regulated phosphoprotein of 32 kDa (DARP32) in the nucleus accumbens and dorsal striatum [45].

The GABA_B_ PAMs were also shown to have beneficial effects in alcohol consumption in rats. Like baclofen, CGP7930 or GS39783 reduced ethanol drinking behavior in two types of inbred alcohol-preferring rats [46, 60]. Both acquisition and maintenance of alcohol dependence were largely inhibited by PAMs, similarly to baclofen.

These first data reveal that GABA_B_ PAMs represent a novel therapeutic strategy for the treatment of drug addiction, a strategy that will certainly benefit from the anxiolytic activity of these molecules. 

## CONCLUSION

Although drugs activating the GABA_B_ receptor were found to have a number of possible therapeutic actions, these were limited because of tolerance and undesired side effects which include sedation, myorelaxing activity and hypothermia. By only enhancing the activity of GABA_B_ receptors when and where needed, the GABA_B_ PAMs respect the physiological activity of the receptor (Fig. ** 5**). Not surprisingly, PAMs were found to have different behavioral effects than the pure agonist baclofen. These molecules lack the undesired side effects of baclofen, can be used in long-term treatment without tolerance, display a more pronounced anxiolytic activity, and show similar positive effects as baclofen in drug addiction. These observations make these modulators excellent alternatives to baclofen for a number of therapeutic applications. 

These recent findings on the GABA_B_ receptor nicely illustrate the power of allosteric enhancers compared to agonists. After the benzodiazepines acting as PAMs at the GABA_A_ receptors, these data represent certainly the second best example of such a class of compounds. A search for similar molecules acting at other receptors is now open.

## Figures and Tables

**Table 1.** Comparison of the Effect and Properties of GABA_B_ Agonists and PAMs

	Agonists	PAMs	Ref.
tolerance	yes	Not after 3 weeks	[44,55]
Body temperature	decrease	No effect	[18]
sedation	increase	No effect	[18]but see [46]
myorelaxation	yes	No effect	[9, 18]
cognition	decrease	No effect	[18]
Anxiety	variable	decrease	[5, 17, 18, 55]
Cocaine self-admin	decrease	decrease	[15, 45, 70, 71]
Alcohol intake	decrease	decrease	[15, 46, 60]
